# Teacher burnout and turnover intention in higher education: The mediating role of job satisfaction and the moderating role of proactive personality

**DOI:** 10.3389/fpsyg.2022.1076277

**Published:** 2022-12-08

**Authors:** Qun Zhang, Xianyin Li, Jeffrey Hugh Gamble

**Affiliations:** ^1^Faculty of Education, Qufu Normal University, Qufu, China; ^2^Chinese Academy of Education Big Data, Qufu Normal University, Qufu, China; ^3^Department of English, National Changhua University, Changhua, Taiwan

**Keywords:** higher education, burnout, turnover intention, job satisfaction, proactive personality, mediation, moderation

## Abstract

**Introduction:**

Teacher burnout and frequent turnover negatively affect stability and productivity in the context of higher education. Despite the fact that the relationship between burnout and turnover intention has been thoroughly studied, the role of other factors in this relationship should be evaluated in order to better clarify underlying mechanisms, particularly in the context of higher education.

**Methods:**

In this study, we first aim to bridge a research gap by utilizing job satisfaction as a mediating variable for the relationship between burnout and turnover intention. Moreover, we uniquely evaluate the role of proactive personality as a moderating variable, first in terms of the relationship between burnout and job satisfaction, and then for the relationship between job satisfaction and turnover intention. Based on 296 valid questionnaires collected from university faculty members in China, proposed hypotheses were evaluated empirically.

**Results:**

The results demonstrate that, as expected, burnout has a significant and positive impact on turnover intention, and job satisfaction has significantly negative impact on turnover intention, with job satisfaction partially mediating the relationship between burnout and turnover intention. Moreover, proactive personality moderated the relationship between job satisfaction and turnover intention, with this relationship being stronger for individuals with high proactive personality as compared to low proactive personality.

**Discussion:**

These findings provide a better understanding of the relationship between burnout and turnover intention of university instructors. Theoretical and practical implications, limitations, and recommendations for further research are provided.

## Introduction

### Factors impacting teacher burnout and turnover in the context of COVID-19

Teacher turnover has been a pressing issue for school administrators for decades. The urgency of turnover in the context of the COVID-19 pandemic has only increased, with research (Matthews et al., 2022) highlighting the importance of administrators’ provision of resources to assist in maintaining teachers’ work-life balance and adaptation to work-related stressors. These stressors include concerns about safety on campus as well as challenges faced by online teaching (Matthews et al., 2022). The role of job satisfaction is one variable of interest in the present study, with low job satisfaction being reported during the pandemic, also demonstrating a significant and negative correlation with the burnout of teachers during COVID-19 among Irish primary school teachers (Minihan et al., 2022). These teachers, as with faculty members in higher education, faced an uncertain environment wherein schools closed and re-opened suddenly, with pressure to teach online without sufficient training and preparation (Minihan et al., 2022).

In the specific context of Chinese university instructors during the peak of COVID-19, job satisfaction was demonstrated to serve as a negative predictor of burnout, as well as a mediator between teacher professional identity (including value, attitude, and sense of belonging components) and burnout (Chen et al., 2020). The teaching and learning environment leading to dissatisfaction for university instructors was characterized by increased workload (including online teaching and technological problem solving) and resulting physiological effects, including insomnia, tension, and fatigue (Chen et al., 2020). Given the importance of job satisfaction, the present study explores the mediating effect of job satisfaction among Chinese university instructors in terms of burnout, as job identity has been considered strongly relevant to burnout, particularly in the context of turnover or job-leaving intention (Kremer and Hofman, 1985).

In terms of burnout during the pandemic, the role of personality has been explored and found to differ significantly among teachers with different “burnout profiles,” with some personality traits (including emotionality—characterized by fear and anxiety in response to life stressors such as family-related stress, economic uncertainty, and abrupt and unpredictable changes to job responsibilities) predictive of burnout among primary and secondary school teachers (Răducu and Stănculescu, 2022). Similar patterns are expected for faculty members in higher education, given parallel changes to their teaching and learning environments. In terms of personality, two studies published in 2021 have supported the role of proactivity, including self-regulative and co-regulative strategies, in preventing teacher burnout (Pietarinen et al., 2021; Pyhältö et al., 2021). While the two studies above approached burnout from the perspective of proactive behaviors, from the perspective of transformative leadership, proactivity can involve both innate proactive personality or can serve a set of behaviors which can be promoted by leadership and organizational culture (McCormick et al., 2019). The need for proactivity, particularly during the pandemic, is based on a challenging educational environment that requires teachers to innovate, think creatively, and enhance their pedagogical skills in order to address the needs of students and the demands of their school. Thus, from the perspective of higher education, proactive personality is a powerful and important construct in the context of COVID-19, as it strengthens the quality of learning and self-efficacy among university students (Zheng et al., 2020) and has been shown to predict improved career adaptability among instructors (Wen et al., 2022), with adaptability associated with lower levels of burnout and turnover intention (Liu et al., 2021) during the COVID-19 pandemic. As such, the present study evaluates the potential role of proactive personality in moderating the relationships among the variables of job satisfaction, burnout, and turnover intention.

### Burnout in the context of Chinese higher education

Presently, the overall enrollment rate in Chinese universities has reached over 50%, indicating a trend toward “universal” higher education in China (Luo, 2021). In the context of expanded university enrolments, the fourth industrial revolution, characterized by large-scale digitization, automation, and the expansion of information technology industries both domestically and internationally (Ito, 2019), has required transformations in Chinese higher education. This transformation to the structure and operations of higher education involves reforms which are progress-oriented, holistic, and fundamental in nature (Lu, 2021). As such, strategic efforts have been promoted in order to focus on fundamentals, such as the needs of industry (Jiang, 2022).

One central focus for change in undergraduate education has been reformed to the faculty evaluation system, with performance-based and accountability-oriented academic evaluation systems being widely adopted (Kang, 2004). This quantitative, results-oriented approach to faculty member evaluation focuses on academic research, emphasizing instrumental rationality and utilitarianism, which can be particularly detrimental to the development of younger teachers in higher education (Tian, 2020). As such, stress, and resulting burnout, is becoming a more common phenomenon among university instructors (Unterbrink et al., 2007). The significant pressure faced by university instructors further diminishes job satisfaction, leading to burnout and turnover intention (Tian and Lu, 2017). As such, when instructors suffer from chronic burnout, they not only have increased intention to leave their job, but also face significantly increased risk of physical and mental illness (Burke and Mikkelsen, 2006).

### Turnover intention and turnover behaviors among instructors in Chinese higher education

Teacher turnover has largely been addressed from two main perspectives: (a) an emphasis on improving job satisfaction or (b) a focus on reducing or eliminating factors leading to job burnout (Pruessner et al., 1999; Parker et al., 2006). Instructors in Chinese higher education have gradually become one of the largest groups of teachers globally (Leiter and Maslach, 2003) and have been characterized by frequent and increasing job turnover in recent years, drawing the close attention of scholars and educational administrators (Smith and Worsfold, 2014). In 2015, a report was released which evaluated instructor burnout and turnover intention in Chinese higher education settings, on the basis of 10,734 respondents from Chinese universities and vocational colleges (MyCOS, 2015). The study found that approximately 51% of university instructors and 58% of vocational college instructors reported strong feelings of burnout, with many of them wishing to leave their current schools. In fact, despite relatively low levels of turnover, the past decade has seen an increased focus on teacher turnover in China due to the pressures turnover creates for organizations (Zhang et al., 2010). In a national survey of university faculties, a majority of faculty members, 54.9%, have considered changing schools, 50.8% have considered changing careers, and only 29.7% have never considered leaving their current position (Du and Liu, 2019). College instructor turnover is often related to pursuing work abroad or in more developed areas (Zhang, 2009), with some surveys finding that 44.3% of Chinese college instructors want to leave and that only 27.2% wish to stay at their current job. This finding echoes the turnover intention of teachers in general, based on a study (Liu and Onwuegbuzie, 2012) of middle school teachers in China, where 40.4% indicated that they would leave the profession if they had the opportunity. Private universities, in particular, have reported actual turnover rates of 12% per year in some provinces of China, largely through the attrition of high-quality teachers in search of better working conditions (Zhao, 2013).

High turnover in higher education settings negatively impacts both instructors who remain employed at these institutions, as well as those who are leaving, as they will be required to invest in altering their career paths, adapting to new conditions, and re-establishing their social networks, endeavors which consume considerable resources when starting in a new workplace (Abifarin, 1997; Oni and Fatoki, 2017; Wongtongkam et al., 2017). From the perspective of higher education management, frequent turnover will require substantial human and material capital to overcome the many problems following turnover, including recruiting replacement employees, selecting and hiring suitable candidates, and providing ongoing training (Harris and Ellis, 2018; Ajayi and Olatunji, 2019).

Moreover, resignations impact the “turnover rate” of organizations, which is an important indicator of organizational stability and the organizational identity which characterizes a workplace (Maertz Jr et al., 2007). In this way, high turnover not only affects the morale of other members of the organization, but also increases the costs of investing in developing and training employees, which are included in the management costs of institutes of higher education (Weng and Xi, 2010). Thus, this turnover of organization members leads to an overall loss of human and social capital, resulting in a negative impact on organizational effectiveness (Shin and Jeung, 2019). Conversely, reducing turnover and stabilizing the organizational environments of institutes of higher education are critical strategies for educational administrators in not only improving efficiency and productivity, but also enhancing social capital, such as networking and collaboration. Therefore, it is essential for universities to understand which factors contribute to turnover intention and to discover how, based on these contributing factors, to effectively retain faculty members. The purpose of this study is to evaluate potential variables which may serve as risk or protective factors in terms of turnover.

Our focus on turnover intention is not only due to its effect on instructors’ psychological health, productivity, and work effectiveness, but also because turnover intention is a reliable and strong predictor of turnover behavior, which is difficult to reliably assess in most cross-sectional research (Liu and Onwuegbuzie, 2012). Furthermore, since surveys of turnover behavior involve personal privacy, most studies use turnover intention as a proxy (Liu et al., 2021). Moreover, turnover behavior (attrition) is difficult to detect given the tendency of educators to remain in higher education after leaving their institution (Bunnell and Poole, 2021). Thus, this study embraces turnover intention as a key factor in relation to turnover behavior based on three key principles: ease of measurement, overcoming the barriers of lack of access to turnover behavior data, and the predictive power of the variable (de Oliveira et al., 2019).

Based on the above discussion, this study is designed to evaluate the relationship between burnout and turnover intention among faculty members in higher education from a novel perspective by evaluating the mediating role of job satisfaction and the moderating role of proactive personality on the relationships between burnout and job satisfaction and job satisfaction and turnover intention. Despite several unique attributes of the Chinese higher education context, we contend that the relationships explored among variables related to teacher burnout, turnover, job satisfaction, and proactive personality should apply cross-culturally to instructors in various higher education contexts, particularly given the demands and innovations emerging from the COVID-19 pandemic (Ali, 2020). As such, the theoretical relationships among the variables introduced in this research are believed to be largely generalizable in nature, and are based on a review of the literature that encompasses studies from a variety of cultures and nations.

### Literature review

#### The direct effect of teacher burnout on turnover intention

The concept of burnout was first proposed by Freudenberger (1974), in the context of service industries, and is characterized by the three factors of individual emotional exhaustion, a sense of dehumanization, and diminished personal accomplishment. Burnout, as a deteriorated sense of engagement with one’s job, arises in work contexts when employees consider their jobs as unpleasant, unenjoyable, or meaningless, consequently leading, in most cases, to a lack of productivity which negatively impacts the efficiency of the work environment (Schaufeli et al., 1996). In the literature, burnout has long been regarded as a key factor leading to increased turnover (Rajendran et al., 2020). As such, burnout is positively related to turnover intention, including the field of teaching (Lee et al., 2018), with long-term burnout forcing many university instructors to quit, change schools, or change careers.

As stability is critical for effective and efficient instruction and university management, burnout has received increasing attention in recent years. Work pressures contributing to job burnout are increasingly salient for faculty members facing mandated, high-stakes performance evaluations (Tamini and Kord, 2011; de Lourdes Machado-Taylor et al., 2016; Luo, 2021). Prior to quitting or resigning from a job (turnover), individuals first begin to contemplate leaving before making a decision (i.e., turnover intention). In this process, burnout serves as a primary risk factor leading to turnover intention and, in many cases, the decision to quit their job (turnover). Therefore, as Friedman (Friedman, 2000) noted, the most serious consequence of burnout is employees’ actual departure from the workplace. Due to the close proximity of intentions to perform a behavior to the behavior itself, scholars have shown that turnover intention is the most predictive variable in terms of actual turnover behaviors (Mobley et al., 1979) a relationship which experts from many fields have confirmed as being typical in the employee turnover process (Cotton and Tuttle, 1986; Griffeth et al., 2000; Batt and Valcour, 2003).

To clarify how burnout relates to turnover intention, it is necessary to concentrate on not only the features of burnout but also the characteristics of turnover intention which can be objectively linked to burnout. Turnover intention reflects the thoughts and decision-making processes of an individual with an organization which have the potential to cause a turnover behavior, resulting in the individual leaving the organization to which they originally belonged and served (Cavanagh and Coffin, 1992). Turnover intention is influenced by internal and external factors, such as employee network groups and policies that enhance employee social embeddedness within the workplace environment, and may be the result of the development of burnout (Friedman and Holtom, 2002). Several studies have supported the positive correlation between burnout and turnover intention from both theoretical and empirical perspectives (Kim and Stoner, 2008; Lee and Lee, 2011). For example, employee burnout has been linked to both poor performance and high turnover rate, such that the higher the level of employee burnout, the lower the performance of the organization overall, with a resulting increase in employee turnover intention leading to higher turnover rates (Cordes and Dougherty, 1993). Other studies have established a positive correlation between burnout and turnover intention from different perspectives, such as the influence of demographic and work characteristics (including gender, age, position, and length of time in the profession) on burnout (Chan et al., 2015) and the influence of individual (personality and work-related attitudes) and organizational characteristics (including workload, control, reward, community, fairness, and values) on burnout and turnover intention (Young, 2015). Data from information technology staff members further support the significant power of the emotional exhaustion dimension of burnout in predicting turnover intention, with the emotional exhaustion and dehumanization dimensions of burnout significantly and positively correlated with turnover intention (Moore, 2000). These findings provide empirical support for the significant effect of burnout on turnover intention. Based on these findings, we propose the following hypothesis:

*H1*: Burnout is positively associated with turnover intention.

#### Mediating role of job satisfaction

Job satisfaction refers to an individual’s feelings or attitudes about their work itself and other aspects related to the work and workplace (Porter et al., 1974). The factors influencing job satisfaction, particularly external factors such as salary, benefits, and interpersonal relationships have been evaluated by several researchers (Dave and Raval, 2014; Özbağ et al., 2014). While these positive, protective factors can increase job satisfaction, negative (risk) factors, such as burnout, are clearly linked to lower levels of job satisfaction (Boamah et al., 2017). Research specifically related to teachers has also demonstrated the significant and negative impact of burnout on job satisfaction. In fact, a study of 563 Norwegian primary school teachers, evaluating several school context variables in addition to teacher burnout and job satisfaction, found that burnout dimensions (particularly emotional exhaustion and reduced accomplishment) were strongly and negatively associated with teachers’ job satisfaction (Skaalvik and Skaalvik, 2009). Studies have shown that the stress and work overload associated with teacher burnout are linked to lower job satisfaction among teachers, while other teacher burnout indicators, such as pressure and anxiety, also served as significant predictors of poor job satisfaction among primary and secondary school teachers in the United States (Liu and Ramsey, 2008). Given the general findings above, in addition to research on teachers at the primary and secondary level, we hypothesize that job satisfaction will be negatively associated with burnout among instructors in higher education.

In terms of turnover, job satisfaction is a measure frequently used by organizations due to its ability to predict a variety of both positive and negative work behaviors, including absenteeism, turnover, and low productivity; for example, among primary care workers (Liu and Ramsey, 2008). Early studies found that low levels of engagement at work, or job satisfaction, could increase faculty member turnover intention for polytechnic (post-secondary vocational school) instructors in the UK (Niven and Cutler, 1995). In fact, meta-analysis of job satisfaction data has revealed that an individual’s level of job satisfaction affects sense of identity and belongingness to the organization, which can influence job performance in addition to both burnout and turnover intention (Judge et al., 2001). According to research with instructors in private institutions of higher education in China (Jiang and Huang, 2020), there is a significant and positive correlation between job satisfaction and the desire to remain at their school, with job satisfaction partially mediating this relationship. Additional research from application-oriented (or vocational) universities in China found that job satisfaction was associated with low levels of turnover intention (Huang and Zhu, 2020). As such, we predict that, in line with the above findings, there will be a direct and negative association of job satisfaction with turnover intention in higher education.

We aim to further evaluate the potential of job satisfaction as a mediator between burnout and turnover intention. Some evidence exists that supports job satisfaction as a mediating variable (Tziner et al., 2015) in terms of work stress, burnout, job satisfaction, and turnover intention among hospital physicians in Israel. In their study, partial mediation was supported by structural equation modeling (SEM), which also demonstrated the negative associations between job satisfaction and both burnout and turnover intention. In fact, in the context of Chinese primary health care, recent studies have found partial mediation of job satisfaction on the relationship between burnout and turnover intention, although with low impact values of 7.4% (Chen et al., 2019) and 5.3% (Ran et al., 2020).

Hayes and Rockwood (2020) define mediation as follows: “Variable X’s effect on a second variable Y is said to be mediated by a third variable M if X causally influences M and M in turn causally influences Y. So X influences Y by inducing change in a mediator variable M, which then carries X’s influence on to Y″ (p: 22). From the studies mentioned above, it is clear that burnout (variable X) is negatively associated with job satisfaction (variable M) which, in turn, is negatively associated with turnover intention (variable Y). This study hypothesizes that changes in job satisfaction resulting from burnout will lead to increased turnover intention (a form of mediation). While we expect that this mediating effect can explain some of the influence of burnout on turnover, we also expect a direct influence of burnout on turnover, thus hypothesizing partial mediation rather than full mediation.

Based on the relationships mentioned above (a) between job satisfaction and burnout (Liu and Ramsey, 2008; Skaalvik and Skaalvik, 2009; Boamah et al., 2017) and (b) between job satisfaction and turnover intention (Niven and Cutler, 1995; Huang and Zhu, 2020; Jiang and Huang, 2020), the role of job satisfaction as a mediating variable that clarifies the relationship between teacher burnout and turnover intention is predicted. As a result, we propose the following hypotheses:

As such, the following hypotheses are proposed:

*H2*: Burnout is negatively associated with job satisfaction.

*H3*: Job satisfaction is negatively associated with turnover intention.

*H4*: Job satisfaction partially mediates the relationship between burnout and turnover intention.

#### Moderating role of proactive personality

The construct of proactive personality was first conceptualized by Swietlik (1968). Bateman and Crant (1993) defined the concept as “the relatively stable tendency to effect environmental change” and as a disposition towards taking action to affect the environment, which adopts the belief that individuals can actively influence their environment. Regarded as a special personality construct (Fuller Jr and Marler, 2009), proactive personality is the embodiment of an individual’s willingness and determination. Individuals with more proactive personalities are adept at identifying opportunities and actively adopt suitable behaviors to achieve their goals and, when confronted with resistance from their environment, will take proactive measures to overcome their current difficulties and resist environmental disruptions. Conversely, individuals with less proactive personalities are less able to identify opportunities, passively accept restrictions from their external environment, or allow themselves to be influenced by their external environment (Kuo et al., 2019).

Numerous studies support the influence of employees’ proactive personality on work performance (Rohland et al., 2004). Employees with proactive personalities positively impact their work environment with corresponding positive results in terms of work performance. Proactive individuals identify opportunities, initiate action, and persist in actively pursuing meaningful transformation (Akgunduz et al., 2020). A number of studies (Rohland et al., 2004; Fuller Jr and Marler, 2009; Tims and Bakker, 2010; Kuo et al., 2019) have indicated that individuals with more proactive personalities will be more active in their jobs by searching for opportunities and ways to change the current environment, resulting in a lower level of turnover intention. Turnover intention will be stronger for individuals with less proactive personalities, facing greater pressure and dissatisfaction at work, along with the inability to change the status quo or to identify opportunities in their current environment (Altura et al., 2020). According to Kuo et al. (2019), the relationship between proactive personality and job satisfaction has been regarded as a comprehensive indicator of a proactive work experience. In fact, one mechanism by which proactive personality mitigates job pressure is by affecting teachers’ views of their surrounding environment (Hernández-Sánchez et al., 2020). Thus, higher levels of proactive personality compensate for some demotivating elements of an individual’s job, thereby affecting job satisfaction (Kumar and Shukla, 2019), often indirectly through positive work-related variables such as social support and hope (Wang and Lei, 2021). The role of proactive personality as a moderator has been supported by previous research of Pakistani respondents from a variety of backgrounds, with a clear moderating effect for workplace variables—in this case, the positive variable of perceived organizational support (Maan et al., 2020). Given the tendency of individuals with higher levels of proactive personality to better deal with stressors in their work environment, thereby achieving a stronger sense of accomplishment and satisfaction with their work, a moderating effect for the relationship between burnout and job satisfaction for instructors in higher education will be evaluated by this study.

A relationship between proactive personality and burnout has been reported among primary school teachers in China, whereby a teacher’s proactive personality produces a sense of achievement, thereby reducing burnout (Gan and Zhao, 2010) which, in turn, should reduce turnover intention. There is evidence that, in general, employees with higher levels of proactive personality engage in actions that are helpful to the organization, using social capital, resulting in lower turnover intention (Yang et al., 2011). A university instructor’s job satisfaction affects turnover intention through its influence on job pressure (Zhu and Jiang, 2015) with higher levels of proactive personality tending to strengthen the effect of job satisfaction in reducing turnover intention. In this way, individuals with higher levels of proactive personality may, through increased job satisfaction, reduce turnover intention (Pang and Wen, 2016). The role of proactive personality in terms of turnover intention is complex, with research suggesting that high levels of proactive personality are indirectly beneficial for decreasing turnover (Yang et al., 2011), based on longitudinal data from China. The authors (Yang et al., 2011) suggest further research into intermediary mechanisms which can provide a more descriptive analysis of the role of proactive personality in terms of other work-related variables and turnover. As such, the potential moderating effect of proactivity personality on the relationship between job satisfaction and turnover intention in higher education will also be evaluated by this study.

Despite years of research in the field of employee turnover and retention (Gan and Zhao, 2010; Wang et al., 2011; Bakker et al., 2012; Pang and Wen, 2016), there is a lack of a comprehensive approach to evaluating higher education burnout and turnover intention in terms of the variables of job satisfaction and proactive personality. Among the few and contradictory findings in the literature is a cross-lagged study which found that the factor of proactive personality was positively correlated with turnover intention (Mobley et al., 1979), and a study reporting that proactive personality had a negative correlation with turnover intention (Wang et al., 2011). Other studies involving these factors include the work of Xu and Yang who investigated the main effect of job satisfaction on counter-productive work behavior, as well as the moderating effect of proactive personality in a survey of 149 employees in Beijing (Xu and Yang, 2013).

The mixed results reported in the literature suggest that the relationship between burnout and turnover intention is complex, and may not be as straightforward as initially expected. This is one motivation for the present study to evaluate the nature of the relevance and role of variables, such as proactive personality, for evaluating burnout and turnover intention.

Hayes and Rockwood (2020) define moderation as follows: “A variable’s effect on another is moderated if its size depends on a third variable - a moderator.” (p: 25). From the studies mentioned above (e.g., Yang et al., 2011; Pang and Wen, 2016; Kumar and Shukla, 2019), it appears that a proactive personality can influence the size, or degree of certain factors in our research model, including job satisfaction and turnover intention. This study hypothesizes that the moderator of proactive personality will influence the degree to which burnout negatively impacts job satisfaction and the degree to which job satisfaction impacts turnover intention.

Based on the literature summarized above, we hypothesize that proactive personality can serve as a positively moderating variable for the relationships between (1) job satisfaction and burnout and (2) job satisfaction and turnover intention, as follows:

*H5*: Proactive personality moderates the relationship between burnout and job satisfaction.

*H6*: Proactive personality moderates the relationship between job satisfaction and turnover intention.

### Contribution of the present study

**Figure 1:**
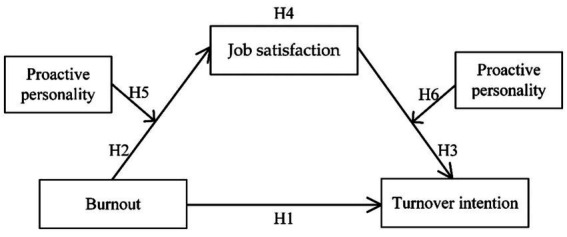
Hypothesized relationships among variables.

This paper aims to bridge the research gap by proposing and testing a series of hypotheses for clarifying the relationship between burnout and turnover intention in the context of higher education. Based on empirical evidence of the relationship between burnout and turnover intention and the partial mediation of job satisfaction, this study contributes to theory by expanding our current understanding of by evaluating the potential of proactive personality as a moderating variable. Moreover, based on an empirical study of instructors from universities in China, we anticipate that our findings can provide deeper insights into the mechanisms involved in burnout and turnover intention among instructors in higher education in order to contribute to the development of informed practice and policy. The hypothesized relationships described above (Section 1.2) are illustrated in Figure 1. While this figure illustrates all hypotheses (including expected mediation and moderation effects), it does not suggest that the entirety of the figure will be tested as a model (i.e., moderated mediation is **not** hypothesized).

The remainder of this paper is organized as follows. Section 2 introduces data collection and analysis procedures; Section 3 provides empirical results for evaluating each of the research hypotheses; Section 4 presents an interpretation of the findings through discussion and conclusions, including specific implications based on the results; and Section 5 includes limitations and areas for future research.

## Materials and methods

### Sample and data collection

For this study, a stratified sampling design was used to collect data from full-time faculty members from colleges and universities in China. Stratified sampling was considered an optimal approach for the collection of data, as it is characterized by the conceptual simplicity. Sampling was based on the assumption that each item of a certain group is given an equal chance to be selected in the sample (Beutler and Leneman, 1966). Stratified sampling was considered a reasonable method for collecting data for this study due to the ability to ensure the estimation accuracy of parameters and representativeness of each sample (Kalton, 2020). Therefore, we uniformly divided universities into five strata based on the 2020 Soft Science Ranking of Chinese Universities (Shanghai Ranking, 2020), which uses hundreds of index variables (including factors such as research output and reputation) to comprehensively evaluate Chinese universities. Two universities were randomly selected from each strata. Random selection of schools from every strata followed the guidelines that (a) all strata were clearly distinguished from other strata and (b) all data within each stratum were consistent, meaning that samples selected from each stratum would be representative of schools in that stratum (Wong and Leung, 1994). Using an online questionnaire, we collected data from each stratum with 33 questionnaires distributed to each university. As such, a total of 330 questionnaires were distributed, with 316 questionnaires collected, for an overall response rate of 95.8%. After removing data from 20 incomplete questionnaires, a total of 296 completed questionnaires were obtained, for an effective response rate of 89.7%. Please refer to Table 1 for details on the data by strata and school.

**Table 1:** Detailed information for stratified sampling.

	University Group (Ranking)					Total
	1 (Top 20%)	2 (Second 20%)	3 (Third 20%)	4 (Fourth 20%)	5 (Bottom 20%)	
Number of schools in each stratum	2	2	2	2	2	10
Number of questionnaires distributed per school	33	33	33	33	33	330
Number of samples collected at per school	31	31	32	32	32	316

Approval was obtained from the institution’s Human Subjects Research committees and pre-notification emails were sent to all participants. One week later, an email was sent containing a link to the online survey questionnaire, information about the research’s purpose, and forms for informed consent. As such, during the process of collecting the surveys, participants were explicitly informed that their responses were anonymous and would not be shared publicly, with the confidentiality and anonymity of the data clearly mentioned. Written consent was obtained from all participants. Demographic data on the composition of the sample are shown in Table 2. In order to evaluate the representativeness of the sample in terms of demographic data, overall population statistics were obtained from the Chinese Ministry of Education (2019). While relatively representative of the population in terms of gender, several differences can be noted. Overall, our sample was younger, with more faculty members possessing doctoral degrees, and more lecturers as compared to professors. Nevertheless, sufficient numbers of observations for each level of each variable were obtained, which was deemed appropriate for the subsequent analysis.

**Table 2:** Demographic data from the sample.

**Variable**	**Classification**	**Number**	**Proportion (%)**	**Overall population of instructors in Chinese higher education: *n* (%)**
Gender	Male	128	43.2	887,780 (48.31%)
	Female	168	56.8	949,834 (51.68%)
Age group	≤ 30	48	16.2	196,892 (10.64%)
	31–40	152	51.4	717,873 (38.76%)
	41–50	80	27.0	557,632 (30.11%)
	≥ 51	16	5.4	379,536 (20.49%)
Education	Bachelor degree	53	17.9	636,608 (34.64%)
	Master degree	112	37.8	687,132 (37.39%)
	Doctoral degree and above	131	44.3	513,874 (27.96%)
Title	Lecturer	112	37.8	193,048 (11.35%)
	Assistant professor	86	29.1	706,607 (41.56%)
	Associate professor	77	26.0	556,711 (32.74%)
	Professor	21	7.1	244,005 (14.35%)
Teaching Experience	≤ 3 years	72	24.3	
	4–8 years	76	25.7	
	9–15 years	86	29.1	Not available
	16–25 years	41	13.9	
	≥26 years	21	7.0	

### Measures

In this study, four variables were measured: burnout, turnover intention, job satisfaction, and proactive personality, based on well-established Chinese versions of measures used widely in the field. All questions in the questionnaire utilized a five-point Likert-type scoring method, with responses ranging from 1 to 5 (1 = “completely untrue” to 5 = “completely true”). A discussion of these measures is provided below.

#### Burnout

The most widely used and validated scale to measure burnout is the Maslach Burnout Inventory—Educators Survey (MBI-ES), compiled by Maslach and Jackson (Maslach and Jackson, 1981). The MBI-ES contains three dimensions with a total of 22 questions. One item for the Emotional Exhaustion dimension includes: “Work makes me feel exhausted.” Dehumanization includes the following item: “I feel very stressed when dealing with students and colleagues.” The Diminished Personal Accomplishment dimension includes the item “Since starting this job, I have become more and more indifferent to people.” Based on the MBI-ES, the wording of the scale was modified and simplified (Li, 2003), with certain items deleted to make the scale suitable for the context of higher education. This process involved translation of the original MBI-ES to Chinese by four expert translators. After reaching consensus, the scale was piloted with 340 individuals from different educational backgrounds, with further refinements made by two language experts and a review by the original authors of the MBI-ES scale (Guo and Xu, 2017). Moreover, data from research in China have established the reliability and validity of the scale. The resulting adapted burnout scale utilized in this study included three dimensions, consisting of a total of 15 items, including five items for Emotional Exhaustion (measured by items 1–5), five items for Dehumanization (measured by items 6–9), and five items for Diminished Personal Accomplishment (measured by items 10–15). The revised scale used for this study demonstrated acceptable reliability overall (Cronbach’s *α* = 0.913). The average variance extracted (AVE) was 0.522, meeting the criteria of AVE > 0.5 (Fornell and Larcker, 1981) indicating acceptable convergent validity.

#### Turnover intention

The four-item scale developed by Farh et al. (1998) was used to evaluate turnover intention. The internal reliability of the scale has been well documented in earlier studies, with Yang (Yang et al., 2005) reporting a reliability coefficient of 0.746, internal consistency of 0.858, and test–retest validity of 0.750. In this study, the scale items were briefly revised (modifying the wording of the scale response anchors to clarify the meaning for Chinese respondents). The resulting internal consistency was Cronbach’s *α* = 0.772 and the split-half coefficient was 0.717, providing strong evidence of scale reliability for the measures of the turnover intention construct. Moreover, the AVE of turnover intention was 0.536, indicating acceptable convergent validity. A sample item includes: “I often think of quitting my current job.”

#### Job satisfaction

To measure job satisfaction, items were adopted from several instruments, including the Minnesota Satisfaction Questionnaire (MSQ) (Weiss et al., 1967), the Job Description Index (JDI) (Gillet and Schwab, 1975), the Job Satisfaction Survey (JSS) (Spector, 1985), and the Employee Satisfaction Survey (SRA) (Bulterys et al., 2006). Teacher job satisfaction was divided into four dimensions: leadership management (measured by items 1–3), compensation management (measured by items 4–6), work itself (measured by items 7–9), and interpersonal relationships (measured by items 10–12). Sample items for the dimensions are as follows: “Various rules and regulations are fair and reasonable” (leadership management), “The actual amount of remuneration is more in line with expectations” (compensation management), “Work can make me realize my potential” (work itself), and “Smooth communication and cooperation with colleagues” (interpersonal relationships). The Cronbach’s α reliability coefficients for each of the four factors belonging to job satisfaction were greater than 0.7 with a reliability coefficient α = 0.913 for the overall scale. Convergent validity was supported since the AVE of the construct was 0.556.

#### Proactive personality

Proactive personality was measured using an adapted version of the Proactive Personality Scale (PPS) originally compiled by Crant (2000). As with other researchers adapting the PPS, this study utilized a shortened scale (Seibert et al., 1999), containing 8 items, which was modified for use with Chinese respondents (Shang and Gan, 2009), on the basis of linguistic and cultural differences. After making slight changes to the wording of the scale, the suitability of the revised version was evaluated, with a split-half coefficient of 0.896 indicating favorable reliability (Cronbach’s *α* = 0.906). The AVE of PPS was ideal, supporting a convergent validity (i.e., 0.605).

### Data analysis

Prior to hypothesis testing, descriptive statistics for target variables (i.e., burnout, job satisfaction, turnover intention, and proactive personality) and discriminant validity were calculated. Correlations among variables, including subscales, are reported in Table 3. In order to support discriminant validity, the square root of the AVEs should exceed the correlation between each pair of latent variables. Subsequently, the results of correlations among observed and latent variables were displayed in order to evaluate the relationships among the variables. Then, to evaluate the predictive nature of these relationships, regression analyses were utilized for testing H1, H2, H3, and H4. We adopted the procedures established by Baron and Kenny (1986) in which different regression models were conducted. More specifically, the relationships between burnout and turnover intention, burnout and job satisfaction, and job satisfaction and turnover intention were examined in models 1, 2, and 3 to address H1, H2, and H3, respectively. In order to evaluate H4, the coefficients of the predictor variables in Models 1, 2, and 3 had to be significant, which is a prerequisite for concluding that job satisfaction served as a mediator between burnout and turnover intention (Baron and Kenny, 1986). In the final model, in which turnover intention was the outcome variable (i.e., Model 4), including burnout and job satisfaction simultaneously, we then compared the coefficient for burnout in Model 1 and Model 4. If the coefficient was changed from significant (in Model 1) to not significant (in Model 4), full mediation would be indicated. If the coefficient was still significant in Model 4 and lower than the value in Model 1, partial mediation would be indicated. To further confirm the results obtained from the comparison among different regression models, we also used a nonparametric bootstrap method for Hayes’ PROCESS macro (Model 4) in SPSS (Hayes and Rockwood, 2020) and directly tested the significance level of the indirect effect of job satisfaction. The PROCESS macro is the most frequently used method to test mediating effects in psychology and other fields and the bootstrap method was deemed more suitable for evaluating the mediating effect test of a small sample data (e.g., our sample) as compared to the Sobel test (Alfons et al., 2018).

**Table 3:** Correlation matrix for all variables and subscales.

	1	2	3	4	5	6	7	8	9	10	11
**1. Overall Burnout**	1										
2. Burnout: Emotional exhaustion	0.842^**^	1									
3. Burnout: Dehumanization	0.812^**^	0.496^**^	1								
4. Burnout: Diminished personal accomplishments	0.899^**^	0.592^**^	0.688^**^	1							
**5. Turnover Intention: overall**	0.732^**^	0.578^**^	0.550^**^	0.726^**^	1						
**6. Job satisfaction: overall**	−0.327^**^	−0.376^**^	−0.190^**^	−0.244^**^	−0.331^**^	1					
7. Job satisfaction: Leadership management	−0.195^**^	−0.282^**^	−0.083	−0.107	−0.186^**^	0.880^**^	1				
8. Job satisfaction: Compensation management	−0.234^**^	−0.281^**^	−0.150^**^	−0.154^**^	−0.225^**^	0.862^**^	0.757^**^	1			
9. Job satisfaction: Work itself	−0.266^**^	−0.293^**^	−0.159^**^	−0.209^**^	−0.284^**^	0.822^**^	0.598^**^	0.585^**^	1		
10. Job satisfaction: Interpersonal relationships	−0.0422^**^	−0.418^**^	−0.262^**^	−0.371^**^	−0.440^**^	0.787^**^	0.565^**^	0.523^**^	0.594^**^	1	
**11. Proactive Personality: overall**	−0.424^**^	−0.384^**^	−0.270^**^	−0.404^**^	−0.380^**^	0.637^**^	0.439^**^	0.465^**^	0.564^**^	0.694^**^	1

*indicates *p* < 0.05; ** indicates *p* < 0.01; *** indicates *p* < 0.001.

Finally, to evaluate H5 and H6, two hierarchical regression models were conducted to examine the possible moderator of proactive personality on a) the relationship between burnout and job satisfaction and b) job satisfaction and turnover intention, respectively. Moreover, Hayes’ PROCESS macro (Model 1) was used to directly test the significance of the moderating effect using the bootstrap method.

## Results

### Descriptive statistics, discriminant validity, and correlation analysis

Table 4 shows the means, standard deviations of observed variables, square root of the AVE of each construct, and correlations among all observed and latent variables. The mean values for the four main factors were burnout (2.136), turnover intention (2.286), job satisfaction (3.440), and proactive personality (3.683). In terms of discriminant validity, since the square root of AVE was greater than the correlations between the inter-latent factors, discriminant validity was supported. Moreover, in line with our theoretical framework, turnover intention was positively correlated with burnout (*r* of observed variables and latent variables were *0*.723 and 0.718, both *p* < 0.01) which, in turn, was significantly and negatively correlated with job satisfaction (*r* of observed variables and latent variables were − 0.331 and − 0.396, *p* < 0.01). Meanwhile, job satisfaction was negatively associated with burnout (*r* of observed variables and latent variables were − 0.324 and − 0.455, *p <* 0.01). Proactive personality also was negatively correlated with both turnover intention (*r* of observed variables and latent variables were − 0.380 and − 0.515, *p <* 0.01) and burnout (*r* of observed variables and latent variables were − 0.416 and-0.518, *p <* 0.01), and was significantly and positively correlated with job satisfaction (*r* of observed variables and latent variables were 0.637 and 0.745, *p <* 0.01).

**Table 4:** Descriptive statistics, square root of the AVE, and correlation analysis among variables.

**Variables**	**Mean**	**S.D**	**Turnover** **intention**	**Burnout**	**Job** **satisfaction**	**Proactive** **personality**
Turnover Intention	2.286	0.807	1/**0.732**			
Burnout	2.136	0.740	0.723**/ 0.718**	1/**0.722**		
Job Satisfaction	3.440	0.735	−0.331**/ −0.396**	−0.324**/ −0.455**	1/**0.746**	
Proactive Personality	3.683	0.664	−0.380**/ −0.515**	−0.416**/ −0.518**	0.637**/ 0.745**	1/**0.778**

*indicates *p* < 0.05; ** indicates *p* < 0.01; the values in bold are the square roots of averaged variance extracted. The underlined values are correlation coefficients between latent variables.

### Regression analysis for testing predicted relationships among variables

Table 5 presents the results of the regression analysis for hypotheses H1, H2, and H3. From the table, the following conclusions can be drawn. First, with burnout as the predictor variable and turnover intention as the outcome variable, the results show that burnout significantly predicts higher turnover intention (*β* = 0.732, *p* < 0.001). Second, with burnout as the predictor variable and job satisfaction as the outcome variable, the results show that burnout significantly predicts lower job satisfaction (*β* = −0.327, *p* < 0.001). Third, with job satisfaction as the predictor variable and turnover intention as the outcome variable, the results show that job satisfaction significantly predicts lower turnover intention (*β* = −0.331, *p* < 0.001). Accordingly, hypotheses H1, H2, and H3 were supported.

**Table 5:** Tests for the mediating effect of Job Satisfaction.

	Turnover intention (Y)	Job satisfaction (M)	Turnover intention (Y)	Turnover intention (Y)
	Model 1	Model 2	Model 3	Model 4
Burnout (X)	0.732***	−0.327		0.699***
Job satisfaction(M)			−0.331***	−0.103***
*R^2^*	0.536	0.107	0.110	0.546
*Adj R^2^*	0.535	0.104	0.107	0.543
*F*	339.944***	35.173***	36.267***	175.985***
df	(1,294)	(1,294)	(1,294)	(2,293)
Hypothesis	H1	H2	H3	
Hypothesis Testing	Supported	Supported	Supported	

*indicates *p* < 0.05; ** indicates *p* < 0.01; *** indicates *p* < 0.001.

Subsequently, given that the coefficients of predictor variables were all significant, we further compared the coefficient of burnout in Model 1 and Model 4. The results showed that the influence from burnout on turnover intention was still significant and the value was reduced from Model 1 (0.732) to Model 4 (0.699). Therefore, job satisfaction served as a partial mediating variable for the relationship between job burnout and turnover intention, and H4 was supported. To confirm the mediating effect of job satisfaction, we also performed nonparametric bootstrap analysis. The results demonstrated that the 95% confidence interval of the indirect effect did not include zero (LLCI = 0.004, ULCI = 0.082), indicating that the mediating effect of job satisfaction was significant.

### Testing the moderating role of proactive personality

To test the moderating role of proactive personality, a set of hierarchical regressions was performed. We first tested H5 with the results of hierarchical regression displayed in Table 6. From the table, we can observe that Model I passed the significance test (*F* = 101.433, *p* < 0.001) and proactive personality had a significant positive impact on job satisfaction (*β* = 0.607, *p* < 0.001). In Model II, proactive personality still had a significant positive impact on job satisfaction (*β* = 0.601, *p* < 0.001). However, the regression coefficient for the interaction term failed to pass the significance test, indicating that proactive personality has no moderating effect on the relationship between burnout and job satisfaction, and thus H5 was not supported.

**Table 6:** Hierarchical regression for testing the moderating effect of Proactive Personality (Predicted Variable: Job Satisfaction).

Variables	Job satisfaction				
	***β***	***R***^***2***^	***F***	***∆R***^***2***^	***∆F***
Model I					
Burnout	−0.069	0.409	101.433^***^	0.409	101.433^***^
Proactive Personality	0.607^***^				
Model II					
Burnout	−0.062	0.413	68.420^***^	0.004	1.824
Proactive Personality	0.601^***^				
Burnout× Proactive Personality	−0.062				

^***^ indicates *p* < 0.001.

Next, H6 was tested. Similarly, a hierarchical regression was conducted and the results are displayed in Table 7. From the table, we can observe that job satisfaction had a significant negative impact on turnover intention in Model I (*β* = −0.150, *p* < 0.05), proactive personality had a significant negative impact on turnover intention (*β* = −0.284, *p* < 0.001), and the model passed the significance test (*F* = 101.433, *p* < 0.001). In Model II, job satisfaction does not significantly predict turnover intention, but proactive personality continues to have a significant and negative impact on turnover intention (*β* = −0.328, *p* < 0.001). Most importantly, the coefficient for the interaction was significant (*β* = −0.154, *p* < 0.01). Moreover, the *F* value of the model was 21.422 at p < 0.001, and was significantly improved compared with Model I (*∆F* = 8.005, *p* < 0.01). This finding provides support for H6.

**Table 7:** Hierarchical regression for testing the moderating effect of Proactive Personality (Predicted Variable: Turnover Intention).

Variables	Turnover Intention				
	***β***	***R***^***2***^	***F***	***∆R***^***2***^	***∆F***
Model I					
Job Satisfaction	−0.150^*^	0.158	27.474^***^	0.158	27.474^***^
Proactive Personality	−0.284^***^				
Model II					
Job Satisfaction	−0.110	0.180	21.422^***^	0.022	8.005^**^
Proactive Personality	−0.328^***^				
Job Satisfaction × Proactive Personality	−0.154^**^				

^*^ indicates *p* < 0.05; ^**^ indicates *p* < 0.01; ^***^ indicates *p* < 0.001.

To more clearly describe the moderating role of proactive personality for the relationship between job satisfaction and turnover intention, we divided proactive personality into low and high proactive personality based on the mean ± 1 SD of proactive personality and adopted the conditional process analysis methods proposed by Hayes and Rockwood (2020). The results showed that for individuals with low proactive personality, there was no significant relationship between job satisfaction and turnover intention while for individuals with high proactive personality, the relationship between job satisfaction and turnover intention was significant (LLCI = −0.404, ULCI = −0.094), demonstrating that proactive personality enhances the impact of job satisfaction on turnover intention.

## Discussion

Amid increasing education system reforms in Chinese colleges and universities, the teaching profession is no longer a “secure job” and the pressures from both parents and students can create emotional labor, which can lead to burnout (Kariou et al., 2021). In China, reforms to the personnel system in colleges and universities have brought about changes in faculty evaluation and intensified competition in the talent market of college instructors, which has increased the awareness of faculty members’ professional crisis, and made pressures in terms of the demands of both scientific research and teaching makes burnout more likely (Smith and Worsfold, 2014). The purpose of this study was to investigate the relationship between burnout and turnover intention by testing the mediating role of job satisfaction between burnout and turnover intention and the moderating role of proactive personality between job satisfaction and turnover intention. Correlation and regression analysis confirmed the hypothesized negative impact of burnout on job satisfaction and the positive, predictive influence of burnout on turnover intention. Based on our analysis, partial mediation of job satisfaction between burnout and turnover intention was supported, as was a significant moderating effect for proactive personality on the relationship between job satisfaction and turnover intention. However, the moderating effect of proactive personality on the relationship between burnout and job satisfaction could not be supported. As such, five of the six hypothesized relationships in our research model were supported by the data.

### The impact of teacher burnout on turnover intention

Reducing or preventing burnout and turnover intention are universally recognized as vital elements for organizational prosperity and sustainability, including educational organization, such as universities (Maertz Jr et al., 2007; Weng and Xi, 2010; Shin and Jeung, 2019). From a review of the current literature review, it is evident that burnout is influential in terms of turnover intention (Lee et al., 2018; Rajendran et al., 2020). Burnout not only has a detrimental impact on the self-development and psychology of teachers but also has adverse consequences on the quality of students’ learning and well-being (García-Carmona et al., 2019; Genoud and Waroux, 2021; Oliveira et al., 2021). Given concerns that burnout and turnover intention are difficult to evaluate and identify (Oliveira et al., 2021), our findings shed light on important aspects of burnout and turnover intention through the empirical modeling and evaluation of data from instructors at Chinese universities. As expected, burnout was strongly predictive of turnover intention, as individuals perceiving a more stressful context will experience higher job turnover intention. This finding echoes those of research studies conducted on teacher burnout and turnover in the United States (Player et al., 2017; Lee et al., 2018).

### The mediating role of job satisfaction in terms of burnout and turnover intention

Job satisfaction, reflecting a psychological state of positivity toward one’s work is also considered as a significant factor in teaching and learning, with evidence demonstrating a strong relationship between job satisfaction and a positive teaching and learning environment, a relationship which is bidirectional (Ortan et al., 2021). While much of the previous research has focused on the theoretical framework of burnout and turnover intention, few studies have examined how burnout affects turnover intention in the context higher education in China. To fill this research gap, this study sought to improve our understanding of burnout and turnover intention in higher education of China by evaluating the mediating role of job satisfaction. Importantly, we demonstrated that job satisfaction is negatively related to job burnout and turnover intention and also serves as a mediating variable. The present study contributes to the literature as few studies have empirically evaluated or modeled the role of job satisfaction in predicting actual burnout and turnover behavior. As such, this study contributes to ongoing scholarship analyzing the relational dynamics that may address positive effects of job satisfaction on burnout and turnover intention (Scanlan and Still, 2019). This research revealed that job satisfaction mediates job burnout and turnover intention, serving to dampen the potential impact of job burnout on turnover intention, in line with previous findings from the manufacturing industry (Pellerone, 2021). However, job satisfaction in teaching is a complex phenomenon and is associated with other variables, such as teacher identity, perceived competence, and rapport with colleagues, which work together to help teachers cope with dehumanizing elements of some aspects of teaching (Atmaca et al., 2020). Moreover, factors such as self-efficacy, in interaction with contextual variables, may indirectly mediate or moderate the relationship between environmental stressors and teacher burnout (Khani and Mirzaee, 2015). As such, the potential of job satisfaction, in addition to related constructs, such as self-efficacy and perceived competence, in mediating burnout and turnover intention are worth further investigation.

Studies have pointed out that low job satisfaction is one of the major risk factors in terms of employees’ susceptibility to burnout (including the three factors of individual emotional exhaustion, dehumanization, and diminished personal accomplishment), finding that emotional exhaustion was the most prominent dimension of burnout (Koeske and Koeske, 1989; Shirom and Ezrachi, 2003; Lee et al., 2017); while personal accomplishment was most strongly associated with job satisfaction (Leiter, 1990). Job satisfaction, therefore, is expected to serve a function in clarifying the relationship between burnout and turnover intention.

### The moderating role of proactive personality

Proactive personality is an important component in evaluating an employee’s performance (Gerhardt et al., 2009), with proactive individuals often actively seeking opportunities and potentials for positive change in their environment, resulting in a lower level of turnover intention (Rohland et al., 2004; Fuller Jr and Marler, 2009; Kuo et al., 2019). In fact proactive personality is positively associated with job satisfaction (Maan et al., 2020) and reduced burnout (Gan and Zhao, 2010) and turnover intention (Zhu and Jiang, 2015). As such, this study tested the moderating effect of proactive personality in terms of job satisfaction and turnover intention in order to contribute to a more complete understanding of the dynamics among the factors of job satisfaction, burnout, and turnover intention. This study contributes to the literature by introducing and testing the potential moderating role of proactive personality between job satisfaction and turnover intention among a population that is vulnerable to high rates of turnover, faculty members in China’s higher education system (Leiter and Maslach, 2003; Smith and Worsfold, 2014). A major contribution of this paper is that we have verified the moderating role of proactive personality on the relationship between job satisfaction and turnover intention in the context of higher education. Moreover, we have found that such moderating effect only exists in faculty members with high proactive personality. We found that, for individuals with high levels of proactive personality, there was a significant and negative effect of job satisfaction on turnover intention, with higher levels of job satisfaction associated with lower turnover intention, suggesting job satisfaction is a decisive factor in turnover for individuals without proactive personality. On the contrary, for individuals with low levels of proactive personality, job satisfaction was not significantly associated with turnover intention.

In fact, proactive personality is significantly and negatively correlated with both burnout and turnover intention and positively and significantly associated with job satisfaction. The implication is that proactive personality may serve to buffer the effects of burnout on turnover intention such that individuals with proactive personalities do not rely upon satisfaction with their job but seek other alternatives to overcome the effects of burnout or other environmental stressors on their psychological well-being and intention to continue teaching. Since previous studies have paid little attention to the role of proactive personality, particularly in terms of burnout and turnover intention, there is little supporting empirical evidence beyond the present study. However, a recent study has found that a proactive personality indirectly affects turnover intention by positively impacting the perceived meaningfulness of one’s job (Rohland et al., 2004). This finding aligns with the moderating effect reported in the present study, as instructors with proactive personalities may overlook factors associated with burnout, including individual emotional exhaustion, sense of dehumanization, and diminished personal accomplishment due to their optimistic belief in the meaningful nature of their job: teaching (Freudenberger, 1974). In sum, this study clarifies the moderating effect of proactive personality on turnover intention for instructors in higher education.

### Implications

This study has identified the significant role burnout plays in turnover intention among Chinese instructors in higher education, the mediating role of job satisfaction in alleviating the effects of burnout on turnover, and the moderating effect of proactive personality in the relationship between job satisfaction and turnover intention. As such, certain implications or contributions may be proposed. In terms of theory, this study contributes to the literature by constructing a model of burnout and turnover intention specific to higher education, which uniquely evaluates both mediation (through job satisfaction) and moderation (in terms of proactive personality). A major finding of this study was that the proactive personality significantly moderated the effect of job satisfaction on turnover tendency, namely that high proactive personality strengthened the relationship between job satisfaction and turnover intention, while job satisfaction was not significantly associated with turnover intention for individuals with low proactive personality. These results can be explained by the fact that, when faced with problems, instructors with high proactive personalities often take initiatives to cope with the impact of environmental changes on their emotions. This finding is in line with previous findings on the relationship between personality and career success (Weng et al., 2016), and extends our understanding of the role of personality in terms of the opposite outcome, career failure, or intention to leave. The findings encourage researchers to consider the interpretive value of both individual personality variables (such as proactive personality) and individual work-related factors (such as job satisfaction). Recent research from Chinese university instructors indicates that job satisfaction is negatively impacted by factors related to balancing teaching and research demands (through emotional exhaustion, an element of burnout), while social and administrative support may increase job satisfaction by increasing engagement (Han et al., 2020). Specific to the higher education context, recent findings from Poland have found that job satisfaction is also positively linked to the significance of their research, scientific opportunities (including teaching and excluding administrative duties), and a sense of passion or achievement in their scientific accomplishments (Szromek and Wolniak, 2020). As such, we believe that the findings from the present study may be applicable across cultures and academic contexts, as instructors in higher education face similar challenges and reward systems (Szromek and Wolniak, 2020).

Next, in terms of implications for practitioners, the finding that proactive personality is associated with lower levels of turnover and higher job satisfaction, such that sufficiently high proactive personality can buffer the effects of burnout on turnover intention, irrespective of an individual’s job satisfaction, suggests that proactive personality is an important and influential factor for instructors. As a stable personality trait (Abid et al., 2021) proactive or other personality traits are not susceptible to training or interventions. However, given the strong association between proactive personality and self-efficacy (Naz et al., 2020), we should consider the relevance of findings related to potential positive influence of offering stability in the educational field, developing a sense of security in attaining educational goals, and reducing external pressures (Barni et al., 2019) in the development of self-efficacy. Other research on teachers, at all levels, has demonstrated that relevant constructs to job satisfaction and proactive personality were instrumental in reducing stress and burnout, particularly the practice of emotional regulation (Carroll et al., 2022).

Other related suggestions include creating a more productive climate, giving more impetus for faculty development, and removing impediments to instructors’ proactive behaviors. As with previous studies focusing on educational leadership (Player et al., 2017), while the relationship between burnout and actual mobility (or turnover) has largely gone unexplored, the impact of school leadership decisions on burnout and turnover intent can be better understood in light of personality factors and job satisfaction, which can be improved through incentives (Altura et al., 2020). Moreover, during the recruitment of faculty members, personality factors, such as proactive personality, may be important in hiring decisions, particularly for schools or positions where burnout is more prevalent. Studies have noted that while not all individuals have an innate proactive personality, two situational elements can be helpful in promoting proactive behaviors: transformative leadership and creating a climate of innovation (McCormick et al., 2019). In terms of transformative leadership, schools can both select and train individuals to lead group sessions (which may involve elements of role-play, providing feedback to peers, and making action plans related to proactive behaviors). In terms of innovation, school culture may be enhanced through the promotion of flexibility and open exchange of ideas, responsive problem solving at the organizational level, and developing awareness of instructors needs among administrators. These initiatives may help to alleviate some of the aspects of rigid performance-based and quantitative evaluation approaches which are known to lead to burnout and turnover (Kang, 2004; Tian, 2020). Specific to the context of COVID-19, some research (Alqassim et al., 2022) has noted the importance of providing incentives (including financial rewards) and an improved environment (with assistance in reducing workloads) for reducing burnout.

From a policy perspective, the implication of our study is that national and local educational authorities, as well as college administrators, should pay attention to the psychological health of college instructors, and consider the importance social support and guidance during the formulation of mental health policies (Friedman and Holtom, 2002; Maan et al., 2020; Wang and Lei, 2021). While maintaining the importance of teaching and research performance, policymakers should also pay attention to the significance of instructors’ personality traits (proactivity) and work-related factors (leading to job satisfaction) when designing professional development, formulating instructor incentive policies, and sensitively enacting examination and evaluation mechanism. Moreover, emphasizing the development of positive personality traits is not only conducive to individual career development, but also to the overall performance of the organization (Liguori et al., 2013). These findings are related to previous findings on the importance of self-efficacy as an element or proactivity, as well as sense of personal accomplishment (Pellerone, 2021) and competence in conducting online teaching, in the context of COVID-19. As such, during the pandemic, the development of autonomy is highly important in preventing burnout and can be facilitated through offering more choices, tailored feedback, support groups, and the provision of other emotional or coping-based counseling (Chang et al., 2022). All in all, while specific guidelines for instructors in higher education have not been widely proposed, elements of successful strategies for teachers at other levels may be applied with some success.

### Limitations and future research

Despite the positive findings and the contributions to both theory and practice, limitations in the present study must be acknowledged. The first of these weaknesses is a relatively small research sample size due to limited access to participants during the pandemic. Although a stratified sampling frame was used in order to select samples from different strata, based on university rank, the small sample size resulted in a sample which was not completely representative of the population overall. As such, future research should attempt to gather more data or adopt an alternative sampling frame, such as simple random sampling. Moreover, the sample was from Chinese instructors in higher education, which somewhat limits generalizations of our research outcomes to other populations with different organizational cultures and expectations. Potential cultural differences in terms of burnout or turnover intention, for example, may exist across higher education contexts globally. As such, future research may evaluate the impact of cultural context on the mediation of job satisfaction and moderation of proactive personality. Additionally, the data were gathered from teaching staff, while non-teaching staff, who also contributes substantially to organizational success, were not surveyed. Finally, the personality trait of proactivity was selected, due to its hypothesized relationship with the other factors in the research model. However, other individual-level or personality factors, including self-efficacy, could be further evaluated in terms of mediating or moderating effects in terms of the relationship between burnout and turnover intention. Finally, while this study has focused on the mediating and moderating effects of job satisfaction and proactive personality, respectively, the unique context of the COVID-19 pandemic has amplified the severity of burnout and turnover. An evaluation of the role of this context on the burnout and turnover intention in higher education is worthy of further study, particularly as teaching and learning evolve in the post-pandemic era.

## Data availability statement

The raw data supporting the conclusions of this article will be made available by the authors, without undue reservation.

## Ethics statement

As the study involved human participants, the research was reviewed and approved by the Institutional Review Board of Qufu Normal University (code 2020005 and 2020/2/19). The study was conducted according to the guidelines of the Declaration of Helsinki and participants provided their written informed consent to participate in this study.

## Author contributions

XL was responsible for supervision and investigation for this research, developing the methodology and validating the results, provision of resources, and acquisition of funding. QZ was involved in the conceptualization of the project, formal statistical analysis, provision of resources, and the drafting and review of the manuscript. JG was involved in the conceptualization and supervision of the project, drafting and review of the manuscript, and visualization of the research results. All authors contributed to the article and approved the submitted version.

## Funding

This research was funded by the National Social Science Foundation of China pedagogy General Project “Research on the School-Running Mode with Mixed Ownership in Vocational Education” (BJA210105).

## Conflict of interest

The authors declare that the research was conducted in the absence of any commercial or financial relationships that could be construed as a potential conflict of interest.

## Publisher’s note

All claims expressed in this article are solely those of the authors and do not necessarily represent those of their affiliated organizations, or those of the publisher, the editors and the reviewers. Any product that may be evaluated in this article, or claim that may be made by its manufacturer, is not guaranteed or endorsed by the publisher.
