# Corrigendum: Teacher burnout and turnover intention in higher education: The mediating role of job satisfaction and the moderating role of proactive personality

**DOI:** 10.3389/fpsyg.2023.1178074

**Published:** 2023-04-03

**Authors:** Qun Zhang, Xianyin Li, Jeffrey Hugh Gamble

**Affiliations:** ^1^Faculty of Education, Qufu Normal University, Qufu, China; ^2^Chinese Academy of Education Big Data, Qufu Normal University, Qufu, China; ^3^Department of English, National Changhua University, Changhua, Taiwan

**Keywords:** higher education, burnout, turnover intention, job satisfaction, proactive personality, mediation, moderation

In the published article, there was an error in the author list. Author “Qun Zhang” was erroneously listed as the second author instead of as the first author. The corrected author list appears below.

“Qun Zhang, Xianyin Li and Jeffrey Hugh Gamble”

Author “Qun Zhang” was also erroneously listed as the corresponding author. Instead, author “Jeffrey Hugh Gamble” should be the only corresponding author.

The authors apologize for these errors and state that they do not change the scientific conclusions of the article in any way. The original article has been updated.

